# Simultaneous Determination of Hormonal Residues in Treated Waters Using Ultrahigh Performance Liquid Chromatography-Tandem Mass Spectrometry

**DOI:** 10.1155/2013/210653

**Published:** 2013-03-06

**Authors:** Rayco Guedes-Alonso, Zoraida Sosa-Ferrera, José Juan Santana-Rodríguez

**Affiliations:** Departamento de Química, Universidad de Las Palmas de Gran Canaria, 35017 Las Palmas de Gran Canaria, Spain

## Abstract

In the last years, hormone consumption has increased exponentially. Because of that, hormone compounds are considered emerging pollutants since several studies have determinted their presence in water influents and effluents of wastewater treatment plants (WWTPs). In this study, a quantitative method for the simultaneous determination of oestrogens (estrone, 17**β**-estradiol, estriol, 17**α**-ethinylestradiol, and diethylstilbestrol), androgens (testosterone), and progestogens (norgestrel and megestrol acetate) has been developed to determine these compounds in wastewater samples. Due to the very low concentrations of target compounds in the environment, a solid phase extraction procedure has been optimized and developed to extract and preconcentrate the analytes. Determination and quantification were performed by ultrahigh performance liquid chromatography-tandem mass spectrometry (UHPLC-MS/MS). The method developed presents satisfactory limits of detection (between 0.15 and 9.35 ng*·*L^−1^), good recoveries (between 73 and 90% for the most of compounds), and low relative standard deviations (under 8.4%). Samples from influents and effluents of two wastewater treatment plants of Gran Canaria (Spain) were analyzed using the proposed method, finding several hormones with concentrations ranged from 5 to 300 ng*·*L^−1^.

## 1. Introduction

In general, it is supposed that more than 100,000 different chemical compounds can be introduced in the Environment, many of them in very small quantity. However, a lot of these compounds are not included as pollutants in the legislation. Although these compounds, named emerging pollutants, are not regulated as pollutants, they probably will be in the future because of their potential negative effect in the ecosystem. For 20 years, many articles have reported the presence of these “new compounds” in wastewater [1, 2].

The emerging pollutant origin is mainly anthropogenic, considering that the majority of these compounds are biologically active substances that are synthesized to use them in agriculture, industry, and medicine. The main source of these emerging pollutants is the residual urban waters and the wastewater treatment plants effluents because many of these WWTPs are not designed or optimized to treat this kind of compounds [3].

Hormones are one of the most potent endocrine disrupting compounds as well as are considered also as emerging pollutants. Hormones can be differentiated in oestrogens, androgens, and progestogens. Some of them have limits in their use, but not a specific legislation [4].

The main characteristic of these pollutants is that it is not necessary to remain in the environment to cause negative effects, in view of the fact that their constant introduction in it offsets their removal or degradation [5].

The steroid hormones help controlling the metabolism, inflammations, immunological functions, water and salt balance, sexual development, and the capacity of withstanding illnesses [6]. The term steroid can be used for natural hormones produced by the body as well as for artificially produced medicines that increase the natural steroid effect.

In the last 50 years, the natural and synthetic hormone worldwide consumption has grown, as much as in human medicine as in cattle farming, and they become the most prescribed medicines [7].

A significant quantity of consumed oestrogens leaves the organism through excretions. For example, 17*β*-estradiol (E2) is oxidized rapidly, becoming an estrone (E1) that can turn into estriol afterwards (E3). Besides, the 17*α*-ethinyl estradiol (EE) is excreted as conjugated [8].

With regard to emission sources, in the first place are the wastewater treatment plants (WWTPs) [9], and secondarily, cattle waste such as those leachates from dung and uncontrolled dumping [10]. Several studies made in the WWTPs have reported that the treatment plants are capable of eliminating around 60% of hormones [11–13].

The identification of hormone residues in environment is of special interest because knowledge of these compounds is a requirement to take measures in order to regulate and minimize their environmental impact.

However, measurement of hormone residues is a very difficult task not only due to the difficulty in measuring very low concentration, but also due to a very complexity of the samples. Therefore, use of mass spectrometer (MS) as detector coupled with chromatography techniques has become a powerful method for the analysis of these types of compounds at trace levels [14–17]. Consequently, LC-MS/MS is the principally chosen technique. One of the main advantages of LC-MS/MS is its ability to analyze hormones without derivatization (necessary in GC) or the need of hydrolyze the conjugated form.

Due to low level concentration of these compounds in environmental water, it is necessary to apply an extraction and preconcentration method prior to LC analysis. The most used technique of extraction and preconcentration method for liquid samples is the solid phase extraction (SPE) [18–20].

The objective of this study is to develop a rapid and simple procedure of extraction, preconcentration, and determination of four steroid oestrogens (estrone (E1), 17*β*-estradiol (E2), estriol (E3) and 17*α*-ethinylestradiol (EE)), one non-steroidal oestrogen, the diethylstilbestrol (DES), one androgen, the testosterone (TES) and two synthetic progestogens, norgestrel (NOR) and megestrol acetate (MGA) (Table 1), based on solid phase extraction and ultrahigh performance liquid chromatography-tandem mass spectrometry (SPE-UHPLC-MS/MS). The developed method was applied to the identification and quantification of these compounds in wastewater samples obtained from the influents and effluents of two wastewater treatment plants (WWTPs) of Gran Canaria (Spain). They presented different methods of wastewater treatments: WWTP 1 presented a traditional method based on activated sludge, while WWTP 2 used a membrane bioreactor technique. 

## 2. Materials and Methods

### 2.1. Reagents

All of the hormonal compounds used were purchased from Sigma-Aldrich (Madrid, Spain). Stock solutions containing 1000 mg·L^−1^ of each analyte were prepared by dissolving the compound in methanol, and the solutions were stored in glass-stoppered bottles at 4°C prior to use. Working aqueous standard solutions were prepared daily. Ultrapure water was provided by a Milli-Q system (Millipore, Bedford, MA, USA). HPLC-grade methanol, LC-MS methanol, and LC-MS water as well as the ammonia and the ammonium acetate used to adjust the pH of the mobile phases were obtained from Panreac Química (Barcelona, Spain). 

### 2.2. Sample Collection

Water samples were collected from the effluents of two wastewater treatment plants located in the northern area of Gran Canaria in May and August of 2012. WWTP 1 used a conventional activated sludge treatment system, while WWTP 2 employed a membrane bioreactor treatment system. The samples were collected in 2 L amber glass bottles that were rinsed beforehand with methanol and ultrapure water. Samples were purified through filtration with fibreglass filters and then with 0.65 *μ*m membrane filters (Millipore, Ireland). The samples were stored in the dark at 4°C and extracted within 48 hours.

### 2.3. Instrumentation

For the SPE optimization, the instrument used was an ultrahigh performance liquid chromatography with fluorescence detector (UHPLC-FD) system consisting of an ACQUITY Quaternary Solvent Manager (QSM) used to load samples and wash and recondition the analytical column, an autosampler, a column manager, and a fluorescence detector with excitation and emission wavelengths of 280 and 310 nm, respectively, all from Waters (Madrid, Spain).

The analysis of wastewater samples was performed in a UHPLC-MS/MS system from Waters (Madrid, Spain), similar to the described above, with a 2777 autosampler equipped with a 25 *μ*L syringe and a tray to hold 2 mL vials, and an ACQUITY tandem triple quadrupole (TQD) mass spectrometer with an electrospray ionization (ESI) interface. All Waters components (Madrid, Spain) were controlled using the MassLynx Mass Spectrometry Software. Electrospray ionisation parameters were fixed as follows: the capillary voltage was 3 kV in positive mode, and −2 kV in negative mode, the source temperature was 150°C, the desolvation temperature was 500°C, and the desolvation gas flow rate was 1000 L/hr. Nitrogen was used as the desolvation gas, and argon was employed as the collision gas.

The detailed MS/MS detection parameters for each hormonal compound are presented in Table 2 and were optimised by the direct injection of a 1 mg·L^−1^ standard solution of each analyte into the detector at a flow rate of 10 *μ*L·min^−1^.

### 2.4. Chromatographic Conditions

For the SPE optimization, the analytical column was a 50 mm × 2.1 mm, ACQUITY UHPLC BEH Waters C_18_ column with a particle size of 1.7 *μ*m (Waters Chromatography, Barcelona, Spain) operating at a temperature of 30°C. Analytes separation was carried out employing the following gradient: starting at 55 : 45 (v/v) water : methanol for 1 minute. During 3 minutes, it changed to 50 : 50 (v/v) and stayed for 2.5 minutes more. Finally, came back to initial conditions in 1 minute, and stayed for 1.5 minutes. Therefore, the analysis took 9 minutes at a flow of 0.5 mL·min^−1^.

For the analysis of real samples, a UHPLC-MS/MS system was used. The analytical column was the same, and the mobile phase was water and methanol, adjusted with a buffer consisting in 0.1% v/v ammonia, and 15 mM of ammonium acetate. The analysis was performed in gradient mode at a flow rate of 0.3 mL·min^−1^. The gradient started at 50 : 50 (v/v) mixture of water : methanol, which changed to 25 : 75 (v/v) in 3 minutes, and returned to 50 : 50 in 1 minute more. Finally, the gradient stayed calibrating for another 1.5 minutes more. The sample volume injected was 5 *μ*L.

## 3. Results and Discussion

### 3.1. Optimization of Solid-Phase Extraction (SPE)

There are a number of parameters that affect SPE procedure such as type of sorbent, pH, ionic strength, sample and desorption volumes, and wash step. To optimize these parameters, it used Milli-Q water spiked with a solution of fluorescence oestrogens (estriol, 17*β*-estradiol, and 17*α*-ethinylestradiol) to obtain a final concentration of 250 *μ*g·L^−1^. 

**Figure 1:**
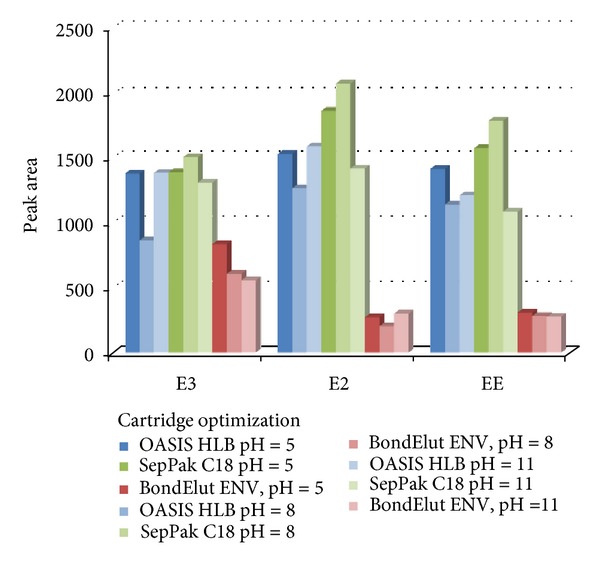
Optimization of SPE cartridges.

The first parameter to optimize is the choice of sorbent, since it controls the selectivity, affinity and capacity over analytes. In this study, the SPE cartridges used were OASIS HLB, SepPak C_18_ (both from Waters, Madrid, Spain), and BondElut ENV (from Agilent, Madrid, Spain). Keeping other parameters fixed (ionic strength of 0%, sample volume of 100 mL), the cartridges were studied at three different pHs (5, 8, and 11). From the results obtained, it can be observed that the better signals are found for SepPak C_18_ cartridge (Figure 1).

**Figure 2:**
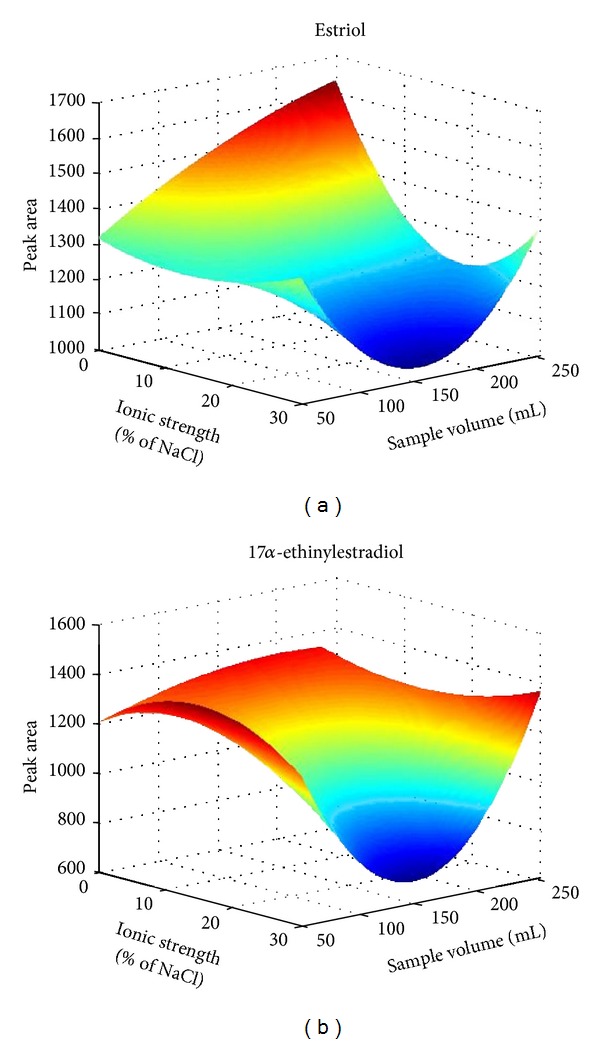
Effect of ionic strength and sample volume on the SPE extraction for estriol and 17*α*-ethinylestradiol.

After choosing the optimum cartridge, we used an initial experimental design of 2^3^, to study the influence of pH, ionic strength and sample volume over extraction process. The experimental design was obtained using Statgraphics Plus software 5.1 and the statistics study was done with IBM SPSS Statistics 19. We assessed two levels and three parameters: pH (3 and 8), ionic strength (0 and 30% of NaCl), and sample volume (50 and 250 mL), to obtain the influence of each parameter and the variable correlation to each other. In this study, it is observed that the ionic strength and sample volume had the major influence on the recoveries of the analytes. For that, a 3^2^ factorial design to optimize these two variables at three levels per parameter (0, 15, and 30% of NaCl for ionic strength and 50, 100 y 250 mL for sample volume) was used.  Figure 2 shows the response surface obtained for the estriol and 17*α*-ethinylestradiol. The results obtained showed that an increment of the ionic strength did not produce an increase in the response area of the compound, and the optimum volume was 250 mL. Because of that, a solution without salt addition and 250 mL of sample volume was chosen. Finally, the desorption volume (1 mL of methanol and 2 mL of methanol in one and two steps) and wash-step (5 mL of Milli-Q water, and 5 mL of Milli-Q water with 5 and 10% of methanol v/v) were assayed to complete the optimization of the SPE process. The optimum values were 2 mL of methanol in one step and 5 mL of Milli-Q water without methanol, respectively.

**Figure 3:**
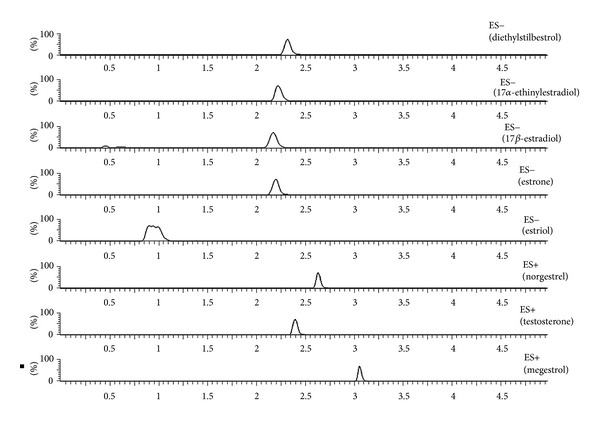
MRM chromatograms of a spiked sample (250 *μ*g·L^−1^) with all analytes after SPE process.

In accordance with the obtained results, the optimum conditions for SPE procedure were SepPak C_18_ cartridge, 250 mL of sample at pH = 8 and 0% of NaCl, desorption with 2 mL of methanol in one step, and wash step with 5 mL of Milli-Q water. In these conditions, we achieved a preconcentration factor of 125. In Figure 3, a chromatogram with the optimum conditions is shown, where the peaks of all compounds in their corresponding transitions can be observed.

### 3.2. Analytical Parameters

Because of the SPE optimization, that was done only with fluorescent compounds (estriol, 17*β*-estradiol, and 17*α*-ethinylestradiol), it was necessary to study the recoveries of all the hormonal compounds, using the optimized SPE-UHPLC-MS/MS method. All the compounds under study showed good recoveries, over 73%, except the diethylstilbestrol, with a recovery of 50.7%.

A calibration curve was used for the quantification of the analytes by diluting the stock solution of each analyte, into the samples to concentrations ranging between 1 and 100 *μ*g·L^−1^. Analysis was conducted by UHPLC-MS/MS and linear calibration plots for each analyte (*r* ^2^ > 0.99) were obtained based on their chromatographic peak areas.

The limit of detection (LOD) and the limit of quantification (LOQ) for each compound were calculated from the signal-to-noise ratio of each individual peak. The LOD was defined as the lowest concentration that gave a signal-to-noise ratio that was greater than 3. The LOQ was defined as the lowest concentration that gave a signal to noise ratio that was greater than 10. The LODs ranged from 0.15 to 9.35 ng·L^−1^ and the LOQs ranged from 0.49 to 31.18 ng·L^−1^.

The performance and reliability of the process were studied by determining the repeatability of the quantification results for all target analytes under the described conditions, using six samples (*n* = 6). The relative standard deviations (RSDs) were lower than 8.4% in all cases, indicating a good repeatability.  Table 3 shows the analytical parameters obtained for all compounds analysed. 

### 3.3. Matrix Effect

Despite the high sensitivity and low chemical noise in UHPLC-MS/MS systems, the sample composition has a great influence on the analyte signal [22]. To evaluate the relative signal enhancement or suppression in the samples, the algorithm by Vieno et al. [23] was used, as following:

(1) $$\begin{array}{c} \frac{\text{As}-\text{(Asp}-\text{Ausp)}}{\text{As}}\times 100, \end{array}$$

where As corresponds to the peak area of the analyte in pure standard solution, Asp to the peak area in the spiked matrix extract, and Ausp to the matrix extract. This procedure was applied to an effluent sample, assuming that all matrices will behave in the same way.

Suppression effect, between 13 and 17%, was observed for estrone, testosterone, norgestrel, and megestrol acetate. For estriol, 17*β*-estradiol, and diethylstilbestrol, the signal suppressions were very low, under 5%. Only 17*α*-ethinylestradiol showed a signal enhancement of 12.6%. The results obtained are showed in Table 3 and they are in accordance with those reported in similar studies [19, 24].

### 3.4. Analysis of Selected Compounds in Wastewater Samples

**Figure 4:**
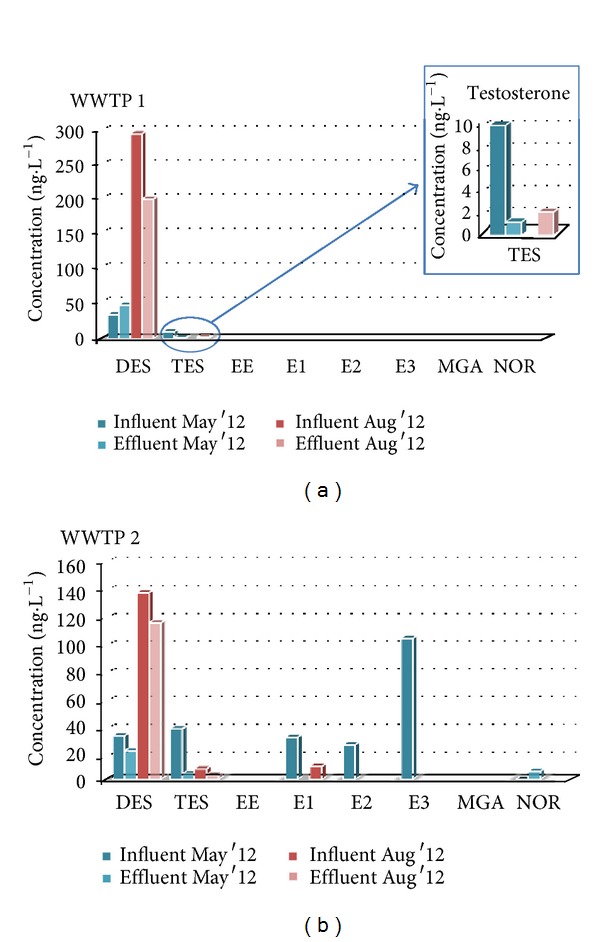
Concentrations of target compounds in sewage samples from both wastewater treatment plants (WWTPs).

To check the efficiency of the developed method, it was applied to determination of target analytes in different wastewater samples from two WWTPs of the island of Gran Canaria (Spain).  Figure 4 shows the results obtained. It can be observed that in the WWTP 1, not all compounds were detected, only diethylstilbestrol and testosterone, in concentration that ranging between 35 and 300 ng·L^−1^ and 1.2 and 9.95 ng·L^−1^, respectively, for influent samples. The concentrations of diethylstilbestrol at the effluent increased in the first sampling and diminished in the second. The behaviour of testosterone in the effluent samples was the opposite.

However, for WWTP 2 a higher number of compounds were detected. For the influents samples, the highest concentrations were between 100 and 140 ng·L^−1^ for estriol and diethylstilbestrol, while the rest of compounds (testosterone, estrone and 17*β*-estradiol) were detected at concentrations between 20 and 40 ng·L^−1^. The concentrations at the effluent for diethylstilbestrol diminished up to 110 ng·L^−1^ and 5 ng·L^−1^ for testosterone. The rest of compounds were not detected at the effluent samples. Only the concentration of norgestrel (about 6 ng·L^−1^) increased during the wastewater treatment process. 

## 4. Conclusions

An analytical method for the simultaneous extraction, preconcentration, and determination of oestrogens (estrone, 17*β*-estradiol, estriol, 17*α*-ethinylestradiol, and diethylstilbestrol) androgens (testosterone) and progestogens (norgestrel and megestrol acetate) in wastewater matrices has been optimized and developed. The method used was solid phase extraction (SPE) for the extraction/preconcentration step and it was combined with UHPLC-MS/MS. The limits of detection reached were between 0.15 to 9.35 ng·L^−1^. In addition, the method presented high recoveries, up to 90%, for the majority of compounds and RSD lower than 9%.

The application of the method to samples from two different WWTPs showed that the concentrations of hormones found, ranged from 5 to 300 ng·L^−1^, and some of them (diethylstilbestrol and testosterone) were detected in all the wastewater samples, and other, like estrone or 17*β*-estradiol, only in some samples. In view of the obtained results about influent and effluent samples, it can be determined that the membrane bioreactor system is quite effective to degrade these compounds. However, it is difficult to obtain a conclusion about the activate sludge treatment effectivity owing to the small quantity of compounds detected.

## Figures and Tables

**Table 1:** List of hormonal compounds, pK_a_ values, chemical structure, and retention times.

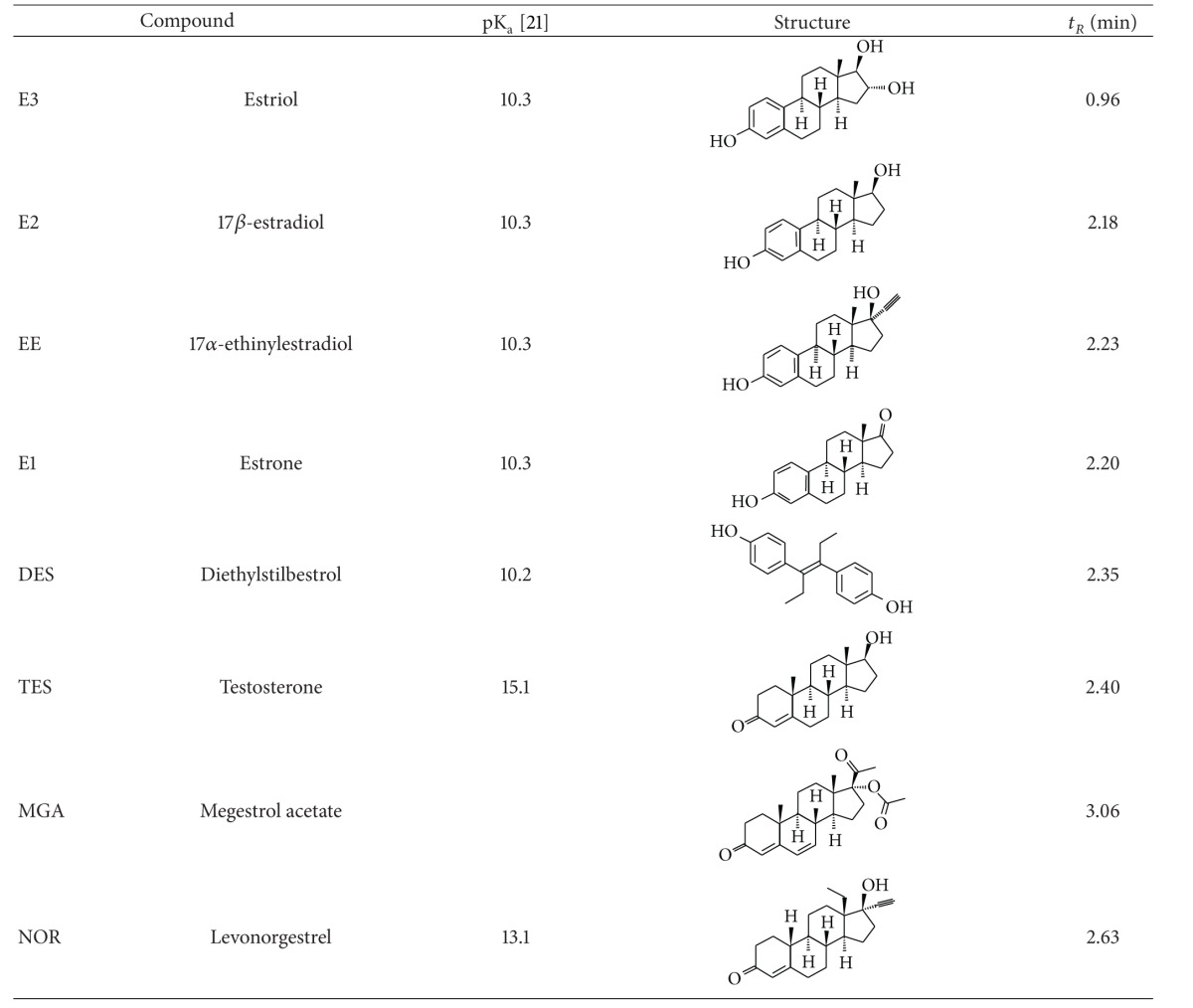

**Table 2:** Mass spectrometer parameters for the determination of target analytes.

Compound	Precursor ion (*m/z*)	Capillary voltage (Ion mode)	Quantification ion, *m/z * (collision potential, V)	Quantification ion, *m/z* (collision potential, V)
E3	287.2	−65 V (ESI −)	171.0 (37)	145.2 (39)
E2	271.2	−65 V (ESI −)	145.1 (40)	183.1 (31)
EE	295.2	−60 V (ESI −)	145.0 (37)	158.9 (33)
E1	269.2	−65 V (ESI −)	145.0 (36)	143.0 (48)
DES	267.1	−50 V (ESI −)	237.1 (29)	251.1 (25)
TES	289.2	38 V (ESI +)	187.0 (18)	104.0 (21)
MGA	385.5	30 V (ESI +)	267.3 (15)	224.2 (30)
NOR	313.2	38 V (ESI +)	109.0 (26)	245.1 (18)

**Table 3:** Analytical parameters for the SPE-UHPLC-MS/MS method.

Compound	RSD^a^ (%) *n* = 6	LOD^b^ (ng · L^−1^)	LOQ^c^ (ng · L^−1^)	Recovery (%) *n* = 6	Matrix effect (%)
E3	6.53	9.35	31.2	80.5 ± 5.3	2.96
E2	8.37	2.53	8.44	89.7 ± 7.5	1.33
EE	7.25	0.51	1.71	90.6 ± 6.5	−12.6
E1	6.81	2.60	8.66	78.7 ± 5.4	15.4
DES	6.93	0.64	2.14	50.7 ± 3.5	4.48
TES	6.77	1.49	4.95	83.8 ± 5.7	17.1
MGA	7.18	0.15	0.49	73.7 ± 5.3	13.5
NOR	7.38	2.11	7.04	88.9 ± 6.5	17.2

^a^Relative standard derivation.

^b^Detection limits, calculated as signal-to-noise ratio of three times.

^c^Quantification limits, calculated as signal-to-noise ratio of ten times.
